# Phacoemulsification and Viscocanalostomy With or Without Silicone Implant in Primary Open-angle Glaucoma: A Randomized Clinical Trial

**DOI:** 10.18502/jovr.v21.17712

**Published:** 2026-02-16

**Authors:** Saeed Shokouhi Rad, Ali Bagheri Asl, Shabnam Niroumand, Hamid Reza Heidarzadeh

**Affiliations:** ^1^Eye Research Center, Mashhad University of Medical Sciences, Mashhad, Iran; ^2^Department of Community Medicine, Faculty of Medicine, Mashhad University of Medical Sciences, Mashhad, Iran

**Keywords:** Glaucoma, Intrascleral Implant, Phacoemulsification, Viscocanalostomy

## Abstract

**Purpose:**

To compare the outcomes of combined phacoemulsification and viscocanalostomy with versus without intrascleral implantation of Ahmed glaucoma valve (AGV) tube remnants in patients with primary open-angle glaucoma (POAG) and cataract.

**Methods:**

A randomized clinical trial involving 60 patients with cataract and POAG was carried out. The case group underwent phacoemulsification and viscocanalostomy together with intrascleral implantation of AGV tube remnants, while the control group underwent the same procedures without any implant. The two groups were compared in terms of best-corrected visual acuity (BCVA), intraocular pressure (IOP), cup-to-disc ratio, number of ocular hypotensive medications, surgical success rate, and scleral lake size.

**Results:**

Sixty patients were enrolled and equally divided into two groups. Postoperative IOP, BCVA, and cup-to-disc ratio did not differ significantly between the groups (*P* = 0.196, *P* = 0.724, and *P* = 0.443, respectively). Despite comparable IOP outcomes, the case group required a significantly higher number of ocular hypotensive medications at the final follow-up (*P* = 0.043). Anterior segment optical coherence tomography (AS-OCT) revealed significantly larger scleral lake dimensions in the case group in terms of length, height, surface area, and perimeter (all *P* ≤ 0.001).

**Conclusion:**

Our study found no significant difference in success rates or postoperative IOP control between combined phacoemulsification and viscocanalostomy with or without silicone implants. Although the case group demonstrated larger scleral lake dimensions on AS-OCT, this anatomical difference did not translate into superior IOP outcomes and was associated with a higher requirement for postoperative ocular hypotensive medications. The clinical significance of increased scleral lake size in the presence of an implant remains uncertain, and longer-term follow-up is warranted.

##  INTRODUCTION 

Non-penetrating glaucoma surgeries, such as viscocanalostomy and deep sclerectomy, which avoid full-thickness penetration of the anterior chamber, reduce intraocular pressure (IOP) with lower complication rates.^[1,2,3]^ However, recent reports suggest that trabeculectomy may be more effective than viscocanalostomy in terms of IOP reduction and success rate.^[4,5]^

Viscocanalostomy is a surgical procedure that uses a microcannula to widen Schlemm's canal by injecting sodium hyaluronate.^[6]^ This helps improve drainage by overcoming the source of outflow resistance, the juxtacanalicular trabecular meshwork.^[7]^ Numerous studies have investigated the efficacy of different intrascleral implants composed of collagen or reticulated hyaluronic acid in preventing the collapse of the intrascleral lake and avoiding fibrosis, thus improving outcomes of deep sclerectomy.^[8,9,10,11,12,13,14]^ However, the results of these studies are inconclusive.

This study aimed to assess the effectiveness of implanting Ahmed glaucoma valve (AGV) tube remnants as a supplementary measure in combined phacoemulsification and viscocanalostomy surgeries performed on patients with primary open-angle glaucoma (POAG) and cataract. We propose that using these implants as spacers in the sub-scleral lake could decrease fibrosis and enhance the long-term success rate of the operation.

##  METHODS

### Study Participants

**Figure 1:**
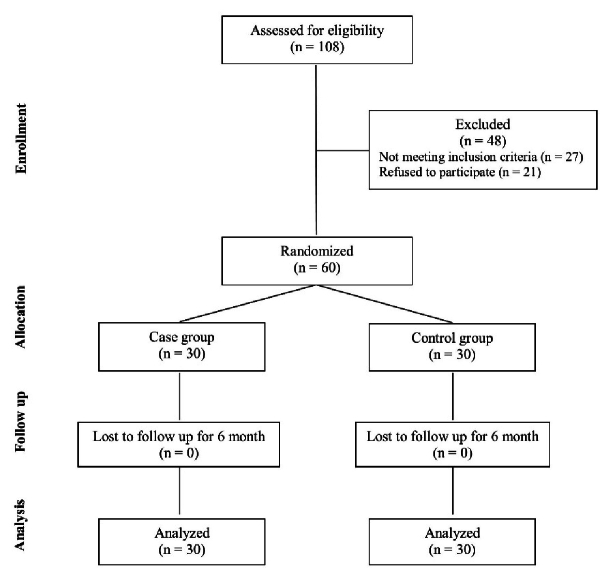
CONSORT flow diagram.

This study was conducted as a randomized clinical trial on 60 adults admitted to the Khatam Eye Hospital (Mashhad, Iran) between 2019 and 2020 who had been diagnosed with cataract and POAG. To participate in the study, individuals had to meet specific criteria, such as providing informed consent and undergoing an ocular examination. We excluded patients who had previously undergone ophthalmic surgery; had moderate or severe axial myopia or hyperopia (over 3 diopters), retinal disease, corneal opacity, strabismus, amblyopia, corneal inflammation, and systemic diseases (including diabetes mellitus, hypertension, autoimmune disease, chronic obstructive pulmonary disease, and sleep apnea); were unable to undergo the required tests; or were unwilling to participate in the study. Patients or the public were not involved in the design, implementation, reporting, or dissemination of this research. Figure 1 demonstrates the CONSORT flow diagram of the trial.

### Study Design

The study comprised two randomly allocated groups of 30 patients. The control group underwent phacoemulsification and viscocanalostomy surgery, while the case group had the same procedures in addition to intrascleral implantation. The randomization scheme was computer-generated using the permuted-block randomization method with varying block lengths of two to six.

**Figure 2:**
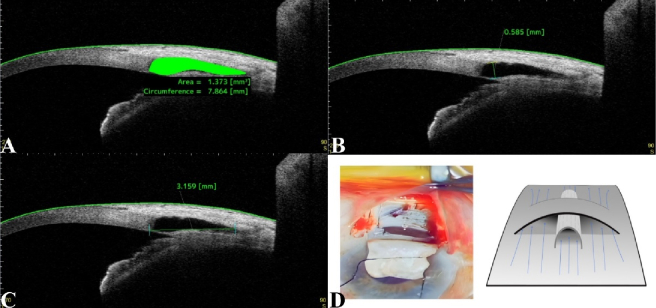
Anterior segment optical coherence tomography (CASIA 2, Tomey Corporation, Nagoya, Japan) for quantitative assessment of scleral lake features. (A) Bleb area and circumference. (B) Bleb height. (C) Bleb length. (D) Intraoperative image and schematic illustration of an AGV tube remnant implant in the scleral lake floor.

Upon admission, a checklist was completed, including the patient's age, gender, IOP, cup-to-disc ratio, number of medications, and best-corrected visual acuity (BCVA). Patients were followed up at 1 week, 1 month, 3 months, and 6 months. IOP, BCVA, and the number of medications were monitored at the first three follow-up visits. At 6 months, in addition to repeating baseline measurements, anterior segment optical coherence tomography (AS-OCT; CASIA 2, Tomey Corporation, Nagoya, Japan) was performed to check the scleral lake features, such as length, height, area, and circumference [Figure 2A–2C].

### Surgical Procedure 

Following phacoemulsification from the temporal side, a traction suture was placed on the cornea using 8-0 nylon. A fornix-based conjunctival flap was created, and light cauterization was performed to achieve hemostasis. A superficial scleral flap measuring 5 × 5 mm and 20-30% scleral thickness was then created, and the dissection was extended 2 mm into the clear cornea. A smaller, deeper flap, measuring 4 × 4 mm, was subsequently fashioned and extended until Schlemm's canal was visible. The eye was decompressed through one of the paracentesis incisions. Then, a blunt dissection was performed using a microsponge to create a Descemet's window that extended over Descemet's membrane and reached 1-2 mm into the clear cornea. The sides of the deep flap were then separated from the adjacent sclera by Vannas' scissors, and the flap was cut. The surgeon removed the juxtacanalicular trabeculum and cannulated both ostia of Schlemm's canal using a cannula on both sides. Subsequently, high-viscosity sodium hyaluronate (Amvisc, Bausch + Lomb, Rochester, NY, USA) was injected into both sides of the canal. Two 10-0 nylon sutures were used in the control group to close the superficial scleral flap tightly. Two additional nylon sutures were then used to secure the conjunctiva.

In the case group, two parallel 2-mm incisions were made in the scleral bed. They were spaced 1.5 mm apart, producing a band-shaped scleral flap. To protect against potential choroidal damage, viscoelastic material was injected into the suprachoroidal space following the initial incision. A remnant silicone tube from a previous AGV surgery was implanted in the suprachoroidal space underneath the band-shaped scleral flap [Figure 2D].^[15]^ A 4-mm tube sterilized with plasma was divided vertically and placed inside the intrascleral space. The surgeries were performed by SSR without any intraoperative complications. After surgery, all patients were given levofloxacin (Levovision-PBAⓇ, Salamat Sazan Pars Bu Ali Pharmaceutical Company, Iran) and betamethasone (BetasonateⓇ, Sina Daru Laboratories, Iran) ophthalmic drops four times daily.

### Statistical Analysis 

The data were analyzed using SPSS version 18.0 (IBM Corp., Chicago, IL, USA). Descriptive statistics, such as mean, standard deviation, median, range, frequency, and percentage, were used to summarize the data. The Kolmogorov–Smirnov test was used to confirm the non-normal distribution of the investigated variables. The paired Student's *t*-test was used as a parametric test for normally distributed data. The Mann–Whitney U test was then used to compare the averages of the variables at 1 week, 1 month, 3 months, and 6 months post-operation. Additionally, the Chi-square test was used to compare qualitative variables between the two groups. A *P*-value of < 0.05 was considered statistically significant.

We performed Kaplan-Meier survival analysis to compare the surgical success rate between the study groups. We defined failure as IOP ≤ 5 mmHg, IOP ≥ 21 mmHg, or the need for ocular hypotensive medication. Also, a Kaplan–Meier curve was constructed. The final sample size was established based on the acceptable levels of type 1 and type 2 errors (alpha and beta) for each research group and the estimated time required to reach this sample size. With an alpha level of 0.05 and a beta level of 0.20 (corresponding to 80% power), a minimum of 30 patients were required in each group. Following the study by Luke et al, we anticipated that a 6-month follow-up period would be necessary to recruit this number of participants.^[12]^

### Ethical Considerations

The clinical trial was conducted in accordance with the Declaration of Helsinki guidelines for the treatment of human participants. Moreover, the study protocol was approved by the Ethics Committee of Mashhad University of Medical Sciences (IR.MUMS.MEDICAL.REC.1398.672) and was registered with the Iranian Registry of Clinical Trials (code: IRCT20200704047997N1). The patients were fully informed about the research objectives and provided written consent.

##  RESULTS 

**Table 1:** Baseline demographic and clinical characteristics of study participants

	**Control group**	**Case group**	***P*** **-value**
Age (Mean ± SD)	70.37 ± 8.63	69.87 ± 9.2	0.833 ${}^{*}$
Females (%)	11 (36.7%)	13 (43.3%)	> 0.999 ${}^{**}$
LogMAR BCVA (Mean ± SD) (Median)	0.63 ± 0.54 (0.61)	0.9 ± 0.75 (0.7)	0.116 ${}^{***}$
Cup-to-disc ratio (Mean ± SD) (Median)	83.17 ± 15.45 (90)	81.83 ± 17.74 (90)	0.757 ${}^{***}$
IOP (Mean ± SD) (Median)	20.9 ± 5.54 (22)	23.27 ± 10.95 (22)	0.295 ${}^{***}$
Number of ocular hypotensive medications (Mean ± SD) (Median)	2.47 ± 0.9 (2)	2.86 ± 0.84 (3)	0.442 ${}^{***}$
Number of patients with IOP > 20 mm Hg (%)	16 (53.3%)	18 (60.0%)	0.821 ${}^{**}$
SD, standard deviation; LogMAR, logarithm of the minimum angle of resolution; BCVA, best-corrected visual acuity; IOP, intraocular pressure; ${}^{*}$ *P*-value based on *t*-test; ${}^{**}$ *P*-value based on Chi-square; ${}^{***}$ *P*-value based on Mann–Whitney U test.			

Sixty individuals with cataract and POAG were randomly divided into two groups, with 30 patients in each group. The mean age of participants was 70.37 ± 8.63 years in the control group and 69.87 ± 9.20 years in the case group. The control group included 11 female participants (36.7%), and the case group had 13 female participants (43.3%). With respect to age and gender, there was no statistically significant difference between the two groups. Table 1 presents baseline information for study participants.

**Figure 3:**
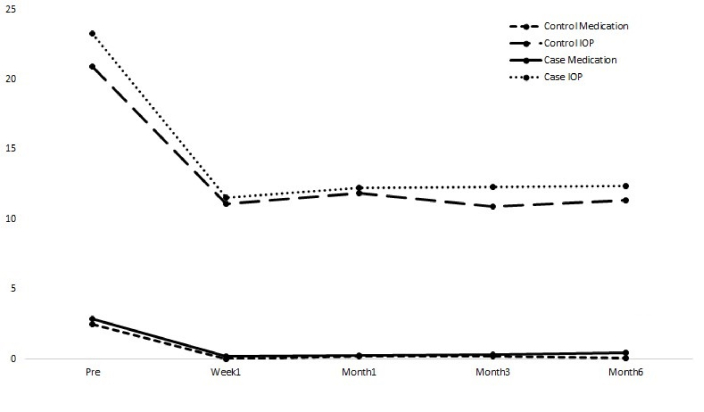
Intraocular pressure (IOP) and the number of antiglaucoma medications in the study groups at baseline and during follow-up visits.

Table 2 presents the mean values of LogMAR (logarithm of the minimum angle of resolution) BCVA, IOP, cup-to-disc ratio, and the number of ocular hypotensive medications for participants at 1 week, 1 month, 3 months, and 6 months post-operation. The mean LogMAR BCVA, IOP, and cup-to-disc ratio showed no significant differences between the two groups in the follow-up visits. Figure 3 shows the mean IOP and the number of ocular hypotensive medications for each study group during postoperative follow-up. During the last follow-up visit, the mean number of ocular hypotensive medications was significantly higher in the case group.

**Table 2:** Clinical characteristics of study participants during follow-up visits

**Follow-up visit**	**LogMAR BCVA (Mean ± SD) (Median)**			**IOP mmHg (Mean ± SD) (Median)**			**Cup-to-disc ratio (Mean ± SD) (Median)**			**Ocular hypotensive medications (Mean ± SD) (Median)**		
	**Controls**	**Cases**	***P*** -value ${}^{*}$	**Controls**	**Cases**	***P*** -value ${}^{*}$	**Controls**	**Cases**	***P*** -value ${}^{*}$	**Controls**	**Cases**	***P*** -value ${}^{*}$
1 week	0.59 ± 0.56 (0.41)	0.72 ± 0.77 (0.4)	0.459	11.1 ± 1.69 (10.5)	11.53 ± 4.53 (10)	0.625	83.17 ± 15.45 (90)	82.17 ± 17.99 (90)	0.818	0 ± 0 (0)	0.17 ± 0.53 (0)	0.091
1 month	0.59 ± 0.56 (0.41)	0.56 ± 0.66 (0.4)	0.883	11.9 ± 3.88 (11.5)	12.24 ± 3.49 (12)	0.724	83.17 ± 15.45 (90)	83 ± 18.6 (90)	0.970	0.2 ± 0.55 (0)	0.27 ± 0.74 (0)	0.694
3 months	0.55 ± 0.53 (0.41)	0.5 ± 0.7 (0.22)	0.755	10.93 ± 2.26 (10.5)	12.31 ± 3.13 (12)	0.060	83.17 ± 15.45 (90)	83.67 ± 17.9 (90)	0.908	0.2 ± 0.61 (0)	0.3 ± 0.83 (0)	0.599
6 months	0.5 ± 0.57 (0.26)	0.45 ± 0.69 (0.22)	0.724	11.33 ± 2.32 (11)	12.38 ± 3.69 (12)	0.196	83.33 ± 15.33 (90)	86.33 ± 14.74 (90)	0.443	0.07 ± 0.36 (0)	0.43 ± 0.89 (0)	0.043
SD, standard deviation; LogMAR, logarithm of the minimum angle of resolution; BCVA, best-corrected visual acuity; IOP, intraocular pressure; ${}^{*}$ Mann–Whitney U test.												

**Figure 4:**
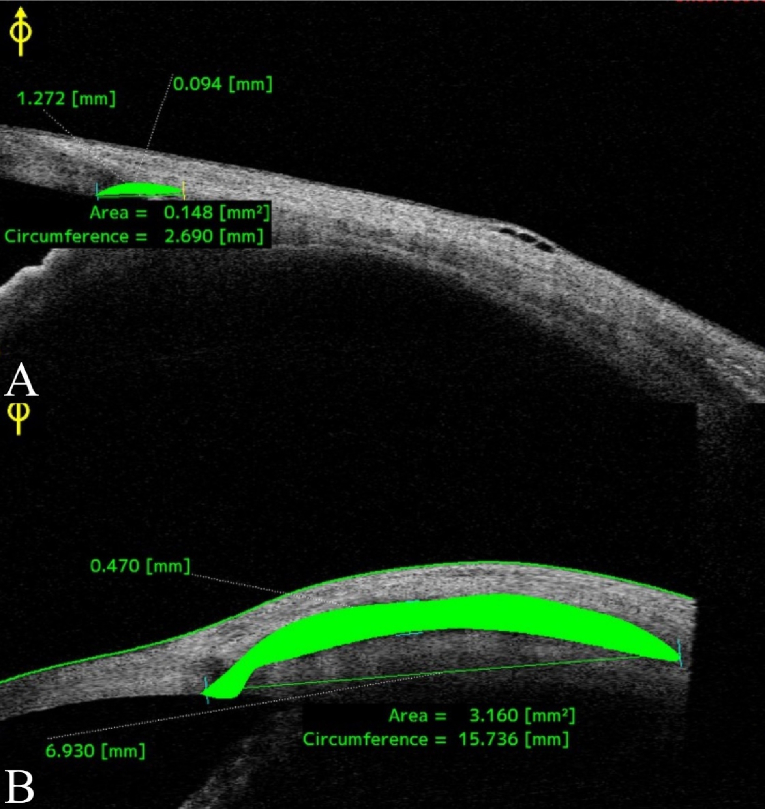
Comparison of bleb features between study groups (A: control, B: case) based on anterior segment optical coherence tomography (CASIA 2, Tomey Corporation, Nagoya, Japan).

Table 3 compares the mean values of the scleral lake's length, height, area, and circumference between the two groups at the 6-month follow-up. The values were notably higher for the group that received the implant [Figure 4].

**Table 3:** Scleral lake features of the study groups evaluated with anterior segment optical coherence tomography (AS-OCT) (CASIA 2, Tomey Corporation, Nagoya, Japan)

	**Controls**		**Cases**		***P*** **-value**
	**Mean ± SD**	**Median**	**Mean ± SD**	**Median**	
Length	1.10 ± 0.69 mm	0.89 mm	4.24 ± 1.16 mm	4.31 mm	< 0.001
Height	0.25 ± 0.23 mm	0.18 mm	0.37 ± 0.15 mm	0.36 mm	< 0.001
Area	0.21 ± 0.23 mm^2^	0.13 mm^2^	1.15 ± 0.44 mm^2^	1.17 mm^2^	0.001
Circumference	2.33 ± 1.59 mm	1.99 mm	9.11 ± 2.48 mm	8.76 mm	< 0.001
SD, Standard deviation; ${}^{*}$ Mann-Whitney U test.					

**Figure 5:**
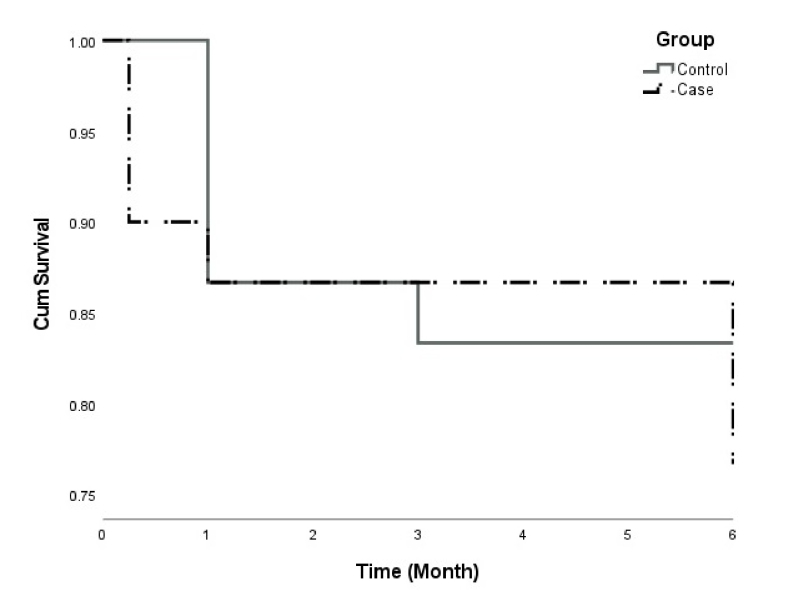
Kaplan–Meier survival analysis curve.

During the 6-month follow-up period, only one patient in the control group had an IOP > 20 mmHg at the first-month follow-up visit. On the other hand, two patients in the case group had an IOP > 20 mmHg during all four post-operation visits. However, this difference was not statistically significant (*P* = 0.0745).

Figure 5 illustrates the Kaplan–Meier survival analysis curve derived from the success criteria outlined in the Methods section. Complete surgical success, as measured by IOP and number of medications, was attained in 83.3% of participants in the control group, compared to 76.7% in the case group. The difference between the two groups was not statistically significant, as indicated by the Log-Rank test (*P* = 0.514).

##  DISCUSSION 

This study assessed the efficacy and safety of using AGV tube remnants as intrascleral implants during phacoemulsification and viscocanalostomy in patients diagnosed with cataract and POAG. Although no statistically significant differences were found between the control and case groups in terms of LogMAR BCVA, IOP, or cup-to-disc ratio during the 6-month follow-up period, the case group demonstrated superior scleral lake morphology. Measurements obtained through AS-OCT indicated that the length, height, area, and circumference of the scleral lake were greater in the case group, key parameters linked to improved aqueous drainage and enhanced long-term surgical success. Furthermore, no complications were associated with the use of the silicone implant.

In 1995, Stegmann introduced viscocanalostomy, a modified type of deep sclerectomy aimed at reducing IOP by excising a portion of the juxtacanalicular trabecular meshwork and inner wall of Schlemm's canal.^[16]^ The procedure widens the canal and creates two openings with viscoelastic materials to drain fluid, allowing aqueous fluid to circulate and exit through the aqueous veins.^[17]^ The juxtacanalicular trabecular meshwork and the inner wall of Schlemm's canal have been identified as the primary impediments to the outflow of aqueous humor.^[18]^ Also, removing 90% of the sclera may create a pathway for aqueous outflow into the suprachoroidal space.^[19]^

In 1989, Fyodorov and Kozlov introduced a collagen implant into the scleral bed, which has been found to enhance the durability of the scleral lake and prevent its collapse.^[8,9]^ Delarive et al reported the development of new drainage vessels in the interscleral space, which absorb aqueous humor flowing to the scleral lake.^[20]^ Therefore, implants protect the residual space from fibrosis and may allow vessels to grow toward the scleral lake. Several studies have confirmed the efficacy of collagen implants in reducing IOP when used as an adjunct to trabeculectomy.^[21,22,23,24]^ A limited number of studies on using collagen implants in conjunction with phacoemulsification and viscocanalostomy have also demonstrated promising outcomes.^[13]^ However, a retrospective analysis has raised doubts about the efficacy of these implants, suggesting that they do not enhance overall results.^[25]^

Deep sclerectomy combined with a reticulated hyaluronic acid implant has shown successful results. In 1999, Sourdille first reported a complete success rate of 72% in his retrospective study.^[11]^ Recent studies have also reported 80% and 57% success rates after the procedure.^[26,27]^ According to a randomized clinical trial conducted by Lüke et al, using a reticulated hyaluronic acid implant in viscocanalostomy resulted in comparable success rates compared to the standard procedure.^[12]^

In viscocanalostomy utilizing mitomycin C (MMC), the agent minimizes scarring and enhances aqueous humor outflow. However, it can also lead to hypotony and other complications.^[28,29,30,31]^ This technique is often analyzed in comparison to trabeculectomy.^[30]^ Viscocanalostomy combined with an intra-scleral implant may produce more predictable outcomes, although it raises concerns regarding the risk of infection and the potential for implant extrusion. A significant aspect of comparison lies in postoperative IOP control and long-term sustainability. While MMC may contribute to reduced fibrosis and improved early-phase results, the intra-scleral implant may offer a more durable drainage mechanism. MMC can result in hypotony and bleb-related complications, whereas an implant may be associated with complications such as infection, erosion, or device migration.^[31,32]^ Our study demonstrated that implants resulted in no complications, at least in the short term. Comparing these two methods in a clinical trial with long-term follow-ups could provide valuable insights.

Our study presents an innovative application of AGV tube remnants as permanent intrascleral implants. These medical-grade silicone implants provide vital structural support to prevent the collapse of the scleral lake and diminish the occurrence of fibrosis. While the case group exhibited notably larger scleral lake dimensions on AS-OCT, this anatomical advantage did not translate into superior short-term clinical outcomes in IOP reduction or medication burden. The case group had a slightly elevated baseline IOP and received more medications, which may suggest a more advanced disease; however, this factor alone is unlikely to account for the observed differences. Another plausible explanation is that the presence of the intrascleral implant, particularly its placement in the suprachoroidal space near the ciliary body, may cause a localized inflammatory response, affecting aqueous production or uveoscleral outflow. These effects may momentarily counterbalance the implant's anatomical benefits. Both groups achieved comparable outcomes; however, the case group displayed enhanced scleral lake anatomy, which is of considerable importance. Notably, no complications or additional financial burdens were incurred by implants.

Importantly, the absence of a statistically significant difference in IOP should not undermine the potential long-term implications of the enhanced lake morphology, as larger scleral lakes may be associated with improved surgical durability.^[13,20]^ These findings warrant cautious interpretation because of the short follow-up duration and modest sample size in the present study. Long-term follow-up is crucial to ascertain whether the structural enhancements observed in the case group result in sustained IOP control and a reduced treatment burden. Furthermore, the structural modifications introduced by the implant may facilitate adjunctive procedures, such as Nd:YAG laser goniopuncture, by preserving a larger scleral lake and stretching Descemet's membrane, thereby making it more approachable for laser treatment.^[13]^

Despite the promising results, the 6-month follow-up period did not reveal significant differences between the two groups in terms of short-term IOP control. This may be attributed to the relatively brief duration of the study, as the benefits of enhanced scleral lake morphology typically become evident over longer follow-up periods. While scleral lake parameters are important surrogate markers for surgical success, longer-term data are essential to determine whether these morphological improvements lead to sustained IOP control and a reduced need for ocular hypotensive medications.

This study has several notable strengths, including its randomized design and rigorous surgical standardization, ensuring a high internal validity. However, it also has significant limitations. The small sample size (*n* = 60) constrains the study's statistical power, making it challenging to identify subtle differences between groups. The brief follow-up period also limits the assessment of long-term outcomes, including scleral lake failure, fibrosis, and late-onset complications. Additionally, the generalizability of the findings to more diverse populations is diminished due to the fact that this study was conducted at a single center and that we excluded patients with systemic diseases or other ophthalmic comorbidities.

In summary, this study offers preliminary evidence that remnants of AGV tubes can be utilized safely and effectively as intrascleral implants during combined phacoemulsification and viscocanalostomy procedures. The improved scleral lake morphology in the case group may suggest potential long-term advantages. However, further research with larger sample sizes, longer follow-up periods, and more diverse patient demographics is crucial to validate these findings and ascertain the full clinical application of this approach. Addressing these limitations in future studies could yield more conclusive evidence to reveal the effects of silicone implants in viscocanalostomy.

##  Financial Support and Sponsorship

The authors thank the Vice-Chancellor of Research at Mashhad University of Medical Sciences for financially supporting this project (code: 971098). The funding organization had no role in the design or execution of this study.

##  Conflicts of Interest

None.
